# The Pharmacist’s Role in Managing COVID-19 in Chronic Kidney Disease Patients: A Review of Existing Strategies and Future Implications

**DOI:** 10.3390/pharmacy10040094

**Published:** 2022-08-05

**Authors:** Mohammed Salim Karattuthodi, Shabeer Ali Thorakkattil, Suhaj Abdulsalim, Sathvik Belagodu Sridhar, Sainul Abideen Parakkal, Savera Arain, Hafees Madathil, Ajmal Karumbaru Kuzhiyil, Mamdouh Mohammed Ahmed Ageeli, Mazhuvanchery Kesavan Unnikrishnan

**Affiliations:** 1 Department of Pharmacy Practice, Manipal College of Pharmaceutical Sciences, Manipal Academy of Higher Education, Manipal 576104, India; 2 Pharmacy Services Department, Johns Hopkins Aramco Healthcare (JHAH), Dhahran 34465, Saudi Arabia; 3 Department of Pharmacy Practice, Unaizah College of Pharmacy, Qassim University, Buraydah 51911, Saudi Arabia; 4 Department of Clinical Pharmacy & Pharmacology, RAK Medical & Health Sciences University, Ras Al Khaimah P.O. Box 11172, United Arab Emirates; 5 Clinical Pharmacist, Saqr Hospital, Ras Al Khaimah P.O. Box 5450, United Arab Emirates; 6 Department of Pharmacy Practice, Nitte Gulabi Shetty Memorial Institute of Pharmaceutical Sciences, Mangalore 575018, India

**Keywords:** COVID-19, chronic kidney disease, pharmacist, patient care

## Abstract

The global burden of the COVID-19 pandemic has not only disrupted healthcare delivery but has also compromised patients’ access to healthcare on account of the scarcity of medications and trained healthcare professionals. COVID-19 has been particularly challenging for patient subpopulations constituting immunocompromised individuals, geriatric patients, and those afflicted by chronic ailments. Reports indicate that diminished kidney function in chronic kidney disease (CKD) renders patients highly susceptible to complications during COVID-19 treatment. Pharmacists, being medication experts, have a significant role in making treatment decisions during COVID-19 infection. This article describes pharmacists’ interventions for monitoring and managing COVID-19 in patients with CKD. Given the massive increase in off-label use of medications to treat COVID-19, pharmacists can contribute substantially towards dosing decisions, reporting adverse medication events, and managing drug–drug interactions in COVID-19 patients suffering from CKD. In addition to traditional methods of delivering their services, the pharmacist should also adopt innovative tele-health systems to optimize patient care and ensure that patients receive safe and effective therapy during the pandemic.

## 1. Introduction

The devastating SARS-CoV-2 infection continues to persist in different parts of the world, expanding the scope of further investigations for both treatment and prevention. By July 2022, the pandemic had infected more than 500 million people and caused 6.4 million deaths [1]. Most importantly, patients with co-morbidities have been more vulnerable to the virus than the healthy population [2]. Chronic kidney disease (CKD) afflicts 10–15% of the global population, who are then at greater risk of contracting COVID-19 [3]. Frequent hospital visits by patients with CKD and end-stage renal disease expose them to the newer variants of the COVID-19 virus. Moreover, studies from China and New York have shown that 30% of COVID-19 patients have had varying degrees of kidney damage. Although the angiotensin-converting enzyme (ACE)-2 receptor (distributed abundantly in the lungs) is the dominant port of entry into human cells, SARS-CoV-2 also penetrates into extrapulmonary organs, causing further damage to the kidneys [4]. The exact mechanism behind renal manifestations in COVID-19 patients is still unclear. Multiple complex pathways could be involved, including direct viral entry into the kidneys and the disruption of the homeostasis maintained by the renin-angiotensin system [5,6]. Nevertheless, severe COVID-19 cases are accompanied by a cytokine storm, leading to systemic inflammation and hypercoagulability, resulting in multiple organ complications that progress to kidney injury [7]. Such patients present high proteinuria, hematuria, elevated serum creatinine, and blood urea, possibly augmented by a diminished oxygen supply [8]. Moreover, mortality from pneumonia is 14–16% higher among CKD patients infected with SARS CoV-19 than in the general population [9]. Providing specialized intensive care for vulnerable patients requires a multidisciplinary approach, involving pharmacists who can suitably assist physicians by selecting the effective therapeutic regimen and individualizing and evaluating recommended therapies, thereby ensuring the optimal utilization of resources [10].

Beyond supplying and dispensing medications, community pharmacists also participate actively in addressing patients’ medication-related issues and promoting the rational use of medications, especially in vulnerable sub-populations, e.g., CKD patients. Community pharmacists are embracing newer roles within tele-pharmacy services, the home delivery of medications, the delivery of timely and reliable drug information services, promoting adherence, and supplying infection preventive items [11].

This article discusses the specialized treatment options for COVID-19 infection in CKD patients, especially those strategies that require dose modification, preventive strategies, enhancing the pharmacists’ role in patient care, and hosting telehealth facilities for assuaging the incidence of COVID-19 infection in CKD patients.

### Role of Pharmacists toward CKD Patients during a Pandemic

COVID-19 infections in CKD patients require multiple interventions by pharmacists, who can play a major role in optimizing medication safety. During the pandemic, pharmacists’ contributions have demonstrated benefits toward improving patients’ health outcomes for chronic conditions, including CKD [12]. Comprehensive assessment of a patient’s medication therapy, offering home delivery and tele-pharmacy, providing appropriate medication information and counseling, identifying and preventing potential medication-related problems, and promoting medication adherence are some of these. Moreover, each patient with pre-dialyzing or dialyzing CKD may require a comprehensive, highly individualized, and variable drug regimen. It is the duty of pharmacists to review each patient’s renal disease burden and suggest medication adjustments accordingly. Pharmacists can contribute significantly to patient care, and the current scenario necessitates the recruitment and training of more community pharmacists to serve CKD patients [13]. Moreover, community pharmacists can promote medication adherence, which is a principal duty in pharmacy practice. Menon and Sander have reported instances of low adherence in low–middle-income countries [14]. Community pharmacists can initiate early interventions with timely reminders, improving medication adherence and proper medication administration, and therefore potentially redefining pharmacists’ roles in chronic illness management [15].

Low–middle-income countries have experienced substantial disruption to medication supplies on account of shortages, factory closures, and lockdowns. In this context, trained pharmacists can substitute out-of-stock drugs with the most appropriate agents available, after verifying this with physicians. This ensures the continuity of therapy for the patient and raises the value of the pharmacy profession [16]. In the long run, promoting competencies in the services of pharmacists would prepare the world for overcoming future healthcare crises.

**Figure 1:**
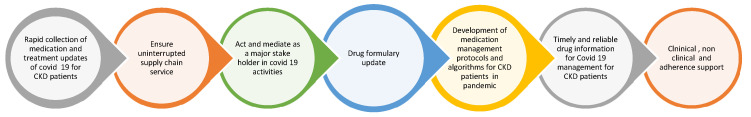
Administrative and dedicated practice-based roles of pharmacists toward CKD patients.

Pharmacists make significant contributions to administrative and practice-based areas of COVID-19 pharmaceutical care (Figure 1). Li et al. proposed a model to optimize the care of CKD patients by creating a physician–pharmacist collaborative agreement that allows nephrologists to refer patients to a pharmacist to optimize their medication management. The practice transitioned into a tele-health model during the COVID-19 pandemic by using the patient portals of electronic health records for the remote blood monitoring of CKD patients. This also resulted in positive outcomes that benefited highly vulnerable patient populations [17]. Schütze et al. reported that medication optimization improved for CKD patients after incorporating a clinical pharmacist in the nephrology clinic in ambulatory care settings [18]. Aghili et al. highlighted the value clinical pharmacists presented by minimizing drug–drug interactions for CKD patients. This clinical pharmacist–prescriber collaboration helps minimize drug interactions and improve the positive therapeutic outcomes in kidney patients [19]. Sub-optimal medication adherence is a significant threat and a great challenge in CKD patients. Islahudin et al. created an innovative prediction model (to check for adherence in CKD) that helps improve medication adherence and therapeutic outcomes for CKD patients [20]. Another study by Liu et al. highlighted the proficiency of the clinical pharmacist in identifying drug-related problems (DRPs) in CKD patients and ensuring their safe and effective use of medications [21].

## 2. Treating COVID-19 in CKD Patients: A Pharmacist’s Perspective

**Figure 2:**
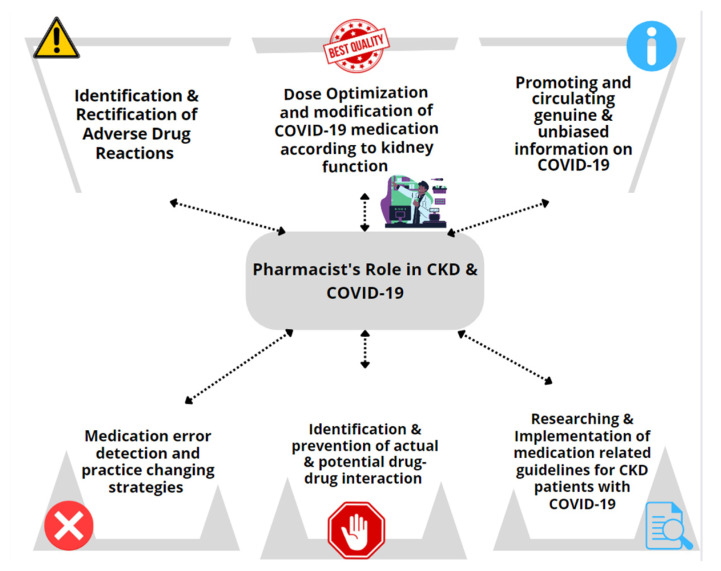
Pharmacists’ clinical role in managing patients with COVID-19 and CKD.

The treatment of COVID-19 patients with underlying CKD needs special consideration. Renal dysfunction can alter the drug’s elimination, thereby causing sub-therapeutic or supra-therapeutic drug concentrations that require close monitoring and/or dose adjustments. While COVID-19 patients are frequently prescribed antivirals, monoclonal antibodies, steroids, and anticoagulants [9,22], determining the renal function of CKD patients is critical for therapeutic success. Consequently, treatment of COVID-19 in patients with CKD requires individualized therapy and a more focused approach from the pharmacist. Figure 2 describes the major clinical roles of pharmacists in treating COVID-19 infection in CKD patients.

The sudden surge in the approval of antivirals for treating COVID-19 has created a knowledge gap around dosing protocols for special populations. Favipiravir is currently recommended at a dose of 1.6 g, twice daily, on the first day, followed by 600 mg, twice daily, for 7 to 14 days. Favipiravir is among the most effective treatment options for COVID-19 infection in end-stage kidney disease (ESKD) and does not require dose adjustments in patients with mild to moderate kidney impairment. However, favipiravir should be avoided when the estimated glomerular filtration rate (eGFR) is <30 mL/min [23]. On account of a dearth of safety information, favipiravir requires close monitoring for the possible persistent rise in serum creatinine (in the absence of other nephrotoxic drugs) and vigorous hemodynamic evaluation in case of acute hypotension, hypertensive emergency, sepsis, and dehydration. If the patient encounters any side effects, then urgent intervention is required via considering dosage adjustments, minimizing the duration of the therapy, or withholding treatment if necessary. Favipiravir attains similar blood concentrations in hemodialysis patients and those with normal kidney function, suggesting its potential use in the CKD population [24]. The Institute for Safe Medication Practice (ISMP) has categorized favipiravir within a list of drugs that have a high risk of causing patient harm if used inappropriately [25]. A pharmacist can play a crucial role in selecting, dosing, and monitoring favipiravir treatment in COVID-19 patients with CKD.

A report from India suggested that remdesivir was well tolerated in patients with kidney damage. Remdesivir does not elevate serum creatinine, even in dialysis patients [26]. Moreover, remdesivir can potentially reduce COVID-19 recovery time, supported by evidence from a clinical trial [27]. In this context, some guidelines recommend that dexamethasone should be co-administrated along with remdesivir. If steroids are contraindicated, baricitnib can be combined with remdesivir. The duration of therapy is restricted to 10 days for severe COVID-19 in a patient, and each hospitalized patient can be treated initially with 200 mg, once, followed by 100 mg, daily, for 5 days. However, in non-hospitalized patients, treatment should be limited to 2 to 3 days [28]. The recommended dose of baricitinib (in patients with CrCl ≥ 60 mL/min) is 4 mg, once daily, orally, as part of a combination regimen for 14 days or until discharge, whichever comes first. The dose should be 2 mg, given orally, once daily, if CrCl is between 30 to <60 mL/min, and is not recommended in patients with CrCl < 15 mL/min or on dialysis because 75% of baricitinib is excreted renaly [29]. Although remdesivir has offered significant benefits to patients with CKD, there are a few concerns. Sulfobutylether beta-cyclodextrin, an excipient in the formulation of remdesvir, can accumulate in patients with impaired kidney function; although, there are no reports of clinically significant adverse effects. The above reason is possibly the motive behind manufacturers not recommending remdesivir when CrCl ≤ 30 mL/min. Remdesivir can be given to selected patients whenever the benefits outweigh the risks, because significant toxicity is unlikely under 5–10 days of treatment. An observational study in ESKD reported an elevation of remdesivir’s metabolite GS-441524 after 5 days of therapy, where approximately 50% was removed through hemodialysis [26,30]. In such situations, pharmacists can contribute to patient care by streamlining dosing schedules and ensuring appropriate therapy and closely monitoring drug–drug interactions, particularly with other nephrotoxic medications.

The Food and Drug Administration (FDA), in December 2021, granted emergency use authorization for molnupiravir for mild–moderate COVID-19 outpatients with a high risk of progression to severe illness. Molnupiravir should be initiated as soon as possible after COVID-19 diagnosis, ideally within 5 days of the onset of symptoms. The recommended dose of molnupiravir is 800 mg, every 12 h, for 5 days. A missed dose can be taken within 10 h from the scheduled time (but not later), with dosing resumed at the next scheduled administration time. Molnupiravir is safe in CKD and does not necessitate dose adjustments [31]. Likewise, nirmatrelvir and ritonavir were approved, yet dose adjustments for their combination are essential when CrCl is <60 mL/min, and contraindicated when CrCl is <15 mL/min [23]. The combination therapy should be initiated at the earliest possible time following a COVID-19 diagnosis, ideally within 5 days of the onset of symptoms. The recommended dose for nirmatrelvir is 300 mg, and for ritonavir is 100 mg, every 12 h for 5 days, when CrCl ≥ 60 mL/min. At a CrCl rate of 30 to <60 mL/min, the dose should be halved (nirmatrelvir to 150 mg and ritonavir to 100 mg) and is not recommended when CrCl < 30 mL/min or in dialysis patients. A missed dose may be taken only if within 8 h from the scheduled time but not after >8 h [32]. Some guidelines propose 300 mg of nirmatrelvir and 100 mg of ritonavir on day 1 and then 150 mg of nirmatrelvir and 100 mg of ritonavir, once daily, for 4 more days when CrCl < 30 mL/min and under dialysis [33]. Pharmacists have a significant responsibility as educators and counselors during the current pandemic.

Certain antiviral drugs with good renal safety profiles should not be considered for COVID-19 because of their limited therapeutic benefit. For example, despite the combination of lopinavir and ritonavir having uncompromised clearance for renal impairment and plasma concentrations unaffected by dialysis, these two antivirals did not improve 28-day mortality or the length of hospital stay, and all reported outcomes similar to standard care (without antiviral) [34]. The National Institute of Health (NIH) COVID-19 treatment guidelines do not recommend lopinavir/ritonavir and other similar agents such as darunavir/cobicistat, azithromycin/chloroquine, and hydroxychloroquine for COVID-19 infection [35].

CKD patients with COVID-19 infections, presenting with mild symptoms for 7 days or less, may be recruited for monoclonal antibody therapy. Sotrovimab, or casirivimab and imdevimab combination, bypass renal elimination and therefore need no dose adjustments. Similarly, tocilizumab, which increases the survival rate of COVID-19 victims, does not require any dose adjustment in mild to moderate renal impairment. However, there are studies in cases of severe renal impairment, creating challenges when administering tocilizumab in ESKD patients [22]. Meanwhile, there are alternatives to tocilizumab, i.e., sarilumab [36] and balmanivimab/etesevimab, reserved for COVID-19 post-exposure prophylaxis and mild to moderate COVID-19 infections, which do not require dose adjustment in renal impairment [37]. Table 1 elaborates on the standard and optimized doses of the most common medications being prescribed for COVID-19.

Corticosteroids are widely prescribed for inflammatory conditions and show a good response. Likewise, steroids suppress the intense phase of inflammation in COVID-19 and the cytokine storm, thereby preventing multi-organ damage. The Randomized Evaluation of COVID-19 Therapy (RECOVERY) trial reported corticosteroid’s potential to reduce mortality in patients requiring oxygenation or ventilation. However, corticosteroids are not considered for mild to moderate infections because of the risk of secondary infections and hyperglycemia. In CKD patients, corticosteroids do not require dosage adjustment even in renal replacement therapy, but patients should be routinely monitored for untoward events [38].

Immunosuppressants, after renal transplantations, can possibly exacerbate COVID-19 complications. A study in pediatric kidney transplanted patients suggested that dose modification or discontinuation of immunosuppressants in COVID-19 positive patients require case-by-case considerations after assessing the risk–benefit ratio [39]. Moreover, although not recommended in CKD patients, NSAIDs may be required during COVID-19 to suppress hyperthermia if the benefit outweighs the risk.

Further, prophylaxis of venous thromboembolism is essential in COVID-19 patients, irrespective of its risk. However, therapeutic modifications may be needed in CKD patients. For those with CrCL > 30 mL/min, anticoagulants, such as unfractionated heparin, enoxaparin, or fondaparinux, may be considered. Besides, a dose reduction label for enoxaparin, and a ‘not recommended’ label for fondaparinux are assigned to those with a CrCl < 30 mL/min [40]. The dose for enoxaparin in patients with CrCl ≥ 30 mL/min is 40 mg SC, once daily, which should be reduced to 30 mg SC, once daily in patients with CrCl < 30 mL/min. It is recommended that the use of enoxaparin be avoided if possible, in patients with dialysis. Moreover, the prophylactic anticoagulant therapy should be discontinued upon discharge or after recovery from COVID-19, when venous thromboembolism risks are resolved [41]. Direct oral anticoagulants are not recommended by chest 2020 guidelines for hospitalized patients due to concerns of an increased risk of bleeding from drug–drug interactions [42]. However, experts consider extended prophylaxis for high-risk patients after discharge, with a direct oral anticoagulant if the CrCl is >30 mL/min, but contraindicated in severe renal failure. The need to select the appropriate anticoagulant and calculate the CrCl and adjust the dose accordingly has widened the scope of pharmacists’ involvement in the treatment of COVID-19 patients with CKD. Communicating and discussing these matters with the physician is of supreme importance, although such practices remain sub-optimal among pharmacists in general.

CKD patients are generally hypertensive and will be on ACE inhibitors or angiotensin receptor blockers (ARBs). No evidence suggests that ACE inhibitors or ARBs exacerbate COVID-19 infections. Unless contra-indicated or substituted with an alternative by the physician, patients are recommended to continue using them [43].

### 2.1. Identification of Adverse Drug Reactions and Drug-Related Errors

COVID-19 has precipitated a healthcare emergency, promoting a number of “off-label prescriptions”. A retrospective observational study at a tertiary care hospital in Malaysia found 246 adverse events in 1080 samples. The highest number of adverse events was reported for the gastrointestinal system (43.5%), followed by the hepatobiliary (36.2%) and cardiac systems (16.3%). The number of adverse events reported was statistically significant and higher in patients with co-morbid conditions, particularly CKD (50%), cardiovascular diseases (39.7%), and those on atazanavir (52.7%), chloroquine (36.8%, and lopinavir/ritonavir (34.6%). While the investigators relied on a trigger tool for adverse event reporting, the pharmacist contributed remarkably to this reporting [44]. This study has reinforced the vital role of pharmacists in pharmacovigilance that promotes medication safety. Similarly, the highest adverse drug event reported by the pharmacist (81.8%) was described in a cross-sectional study in 71 hospitals, particularly for COVID-19 patients in Brazil [45].

Detection, reporting, and formulating a solution for adverse drug reactions and drug-related problems are among the prime responsibilities of a pharmacist. Each event should be justified with evidence from authentic sources. New and vital information generated from such intensive reporting of adverse events can decrease similar occurrences in the future by increasing vigilance, hence augmenting the standard of patient care.

### 2.2. Counteracting Drug-Drug Interactions in COVID-19 Patients with CKD

The rise in the global prevalence of CKD mandates the careful monitoring of potential drug–drug interactions. Impaired kidney function alters drug concentrations in COVID-19 patients, potentially reducing their efficacy or enhancing their toxicity. Concomitant drug–drug interactions and higher doses are acceptable only if the benefits outweigh the risks [46]. For instance, tramadol and remdesivir could precipitate a critical drug–drug interaction, and both medications need dose adjustments in CKD. The combination has the potential for an acute pain crisis, secondary to the drug–drug interaction [47]. Similar events can happen during poly-pharmacy, which is common in patients with CKD diagnosed with COVID-19. This would increase the chances for more drug–drug interactions. A study by Schütze et al. reported improved outcomes in medication optimization in CKD patients after incorporating a clinical pharmacist role in a nephrology clinic in ambulatory care settings [48]. With sound knowledge of both pharmacodynamics and pharmacokinetics, pharmacists can suggest suitable modifications to the frequency, dosage, or drugs to minimize drug–drug interactions and subsequent adverse effects.

### 2.3. Tele-Pharmacy Based Pharmaceutical Care Services for CKD Patients

The COVID-19 pandemic has greatly impacted CKD/ESKD patients, especially those in vulnerable groups, by limiting regular access to health care [49]. Many strategies have emerged for ensuring best practices for safe healthcare delivery. For example, CKD patients who require regular pharmaceutical care during the COVID-19 pandemic can use tele-pharmacy technology to minimize face-to-face interaction [50]. The impact of telepharmacy-based pharmaceutical care services on chronic diseases has been widely researched [51,52]. Johnstone et al. documented 667 clinical pharmacy interventions via tele-pharmacy among CKD patients in Queensland, Australia [48]. Amkreutz et al. reported on the implementation of tele-pharmaceutical expert consultation (in addition to tele-intensive care unit services) and mention 26 recommendations for dosage adjustments in renal failure patients, 11 of which were implemented [53].

Keeys, while highlighting the nighttime pharmaceutical care services in a community hospital in the USA, documented interventions such as dosage adjustment and adverse drug reaction management among patients with renal insufficiency. Another study evaluating medication errors in rural critical access hospitals in the North Dakota Telepharmacy Project (NDTP) documented 182 renal-dosing-related interventions [10]. During the COVID-19 pandemic in Jordan, Hammour et al. reported the delivery of 557 (10.1%) nephrology-related prescriptions from the hospital pharmacy department using an internet-based drug delivery platform model. Similarly, a Spanish tele-healthcare-based study reported erythropoiesis-stimulating agents as the most frequently requested medication via the tele-healthcare services, where 285 CKD and cancer patients requested this medication [54].

Clinical pharmacists employed tele-monitoring services to adjust the drug dosages in a tertiary care hospital in Thailand for COVID-19 patients with severe kidney injury undergoing continuous renal replacement therapy [55]. Another Thailand-based study documented 37 (15.04%) DRPs among 93 (18.64%) CKD patients receiving home drug delivery during the COVID-19 pandemic [56]. A cluster-randomized study, which included CKD patients, documented a statistically significant (*p* < 0.001) decrease in systolic and diastolic blood pressure when patients were provided with intervention via tele-monitoring services.

The above publications underline the inevitable need for tele-pharmacy-based pharmaceutical care services for vulnerable patients. Published research demonstrates that the delivery of a comprehensive range of pharmaceutical care to renal failure patients can be continued only with tele-pharmacy services included. Innovative technology offers boundless opportunity to improve access to pharmaceutical care for patients with CKD or ESKD.

### 2.4. Pharmacists Managed Home Delivery Service for CKD Patients

Patients with co-morbidities are more likely to contract the COVID-19 virus and have worse outcomes. In particular, CKD patients with additional underlying medical conditions are at a significantly higher risk. Based on their risk assessment, it is therefore important to minimize their visits to healthcare facilities to avoid the transmission of the virus. In this situation, the home delivery of medication by pharmacists brings with it commendable advantages. In compliance with the international COVID-19 recommendations for pharmacists and the pharmacy workforce, as well as country-specific COVID-19 guidelines, many pharmacies around the world provide daily home deliveries of drugs, essential supplies, and dialysis consumables [57,58].

### 2.5. Prevention and Management Strategies for Pandemic Related Risks

Chang et al. reported frequent infections in non-dialyzing CKD patients, which increased when eGFR decreases [59]. Similarly, the prevalence of infection can increase with hospital visits and direct patient contact during dialysis. Proper preventative strategies and precautions should be available for CKD patients who avail services in dialysis centers. Streamlined guidelines and recommendations for hand hygiene, isolation precautions, and the timely identification of COVID-19-positive patients are some examples of good practice. Isolation and precautions are essential in dialysis units because multiple patients visit the same dialysis area frequently. There should be a system that aids the early detection of COVID-19 cases that would protect other CKD patients at risk of infection.

## 3. Conclusions

Patients with CKD are at higher risk of developing complications and may face more severe symptoms upon contracting COVID-19 infection. Antivirals, monoclonal antibodies, steroids, anticoagulants, and CKD medications are a part of the standard treatment regimen as per the COVID-19 treatment guidelines and updates issued by health authorities across the globe. While more studies are still underway to measure the impact of COVID-19 on CKD patients, pharmacists can play a major role in identifying, preventing, and managingDRPs, performing dose adjustments based on renal function, and counseling and motivating patients about the importance of medication adherence and lifestyle modifications for preventing complications in CKD. Pharmacists can also act as medication experts through tele-medicine by providing drug information, especially in places where the pandemic has severely restricted COVID-19 patients from hospital access. Pharmacists have really been at the front and center from the very beginning of the situation. Looking ahead, several things are going to change as a result of what pharmacists have done during the crisis, especially the scope for expanding practice and the increased demand for the types of services that pharmacists provide.

## Figures and Tables

**Table 1:** Standard and adjusted doses based on renal impairment for widely utilized medicines against COVID-19.

Sl No.	Medication	Usual Adult Dose	Renal Dose Modification
1	Favipiravir	1.6 g, twice daily, on day 1, followed by 600 mg, twice daily, orally, for 7 to 14 days	**Mild to moderate impairment:** No dose adjustment required **eGFR < 30 mL/min:** avoid **Dialysis:** blood concentration of favipiravir in patients on hemodialysis similar to those with normal kidney function, suggesting it’s use in CKD.
2	Remdesivir	For hospitalized patients: 200 mg on day 1 and 100 mg daily for 5 days or until discharge. For non-hospitalized patients: 200 mg on day 1 and 100 mg on days 2 and 3	**CrCl ≥ 30 mL/min:** No dose adjustment required **CrCl < 30 mL/min:** Manufactures do not recommend remdesivir. Significant toxicity unlikely with short term treatment (5 to 10 days) Dialysis: remdesivir at recommended doses do not normally increase serum creatinine including in patients on dialysis
3	Molnupiravir	800 mg every 12 h for 5 days	No dose adjustment recommended
4	Nirmatrelvir and Ritonavir	**CrCl ≥ 60 mL/min:** Nirmatrelvir 300 mg and ritonavir 100 mg every 12 h for 5 days	**CrCl 30 to <60 mL/min:** nirmatrelvir 150 mg & ritonavir 100 mg, every 12 h for 5 days **CrCl is <30 mL/min:** Not recommended **Dialysis:** Not recommended Some guidelines propose nirmatrelvir 300 mg and ritonavir 100 mg on day 1, then nirmatrelvir 150 mg and ritonavir 100 mg, once daily, for 4 more days when CrCl < 30 mL/min and dialysis (dose after dialysis)
5	Sotrovimab	500 mg IV as a single dose	No dose adjustment recommended
6	Casirivimab and Imdevimab	IV, subcutaneous: casirivimab 600 mg and imdevimab 600 mg as a single dose	No dose adjustment recommended
7	Tocilizumab	IV: 8 mg/kg once, second dose may be considered ≥ 8 h if no clinical improvement	**Mild to moderate impairment:** No dose adjustment recommended **Severe impairment:** No dose adjustment noted in the manufacturer’s label (no data), including in patients on dialysis. But significant renal elimination unlikely because of its molecular weight
8	Sarilumab	400 mg IV once	No dose adjustment recommended
9	Balmanivimab/Etesevimab	Balmanivimab 700 mg, etesevimab 1.4 g, IV single dose	No dose adjustment recommended
10	Baricitinib	**CrCl ≥ 60 mL/min:** 4 mg, once daily, oral, for 14 days	**CrCl 30 to <60 mL/min:** 2 mg, once daily, orally. **CrCl 15 to <30 mL/min:** 1 mg, once daily, orally. **CrCl < 15 mL/min or Hemodialysis:** not recommended

## Data Availability

Not applicable.
